# Widespread White Matter Alterations in Patients With Visual Snow Syndrome

**DOI:** 10.3389/fneur.2021.723805

**Published:** 2021-09-21

**Authors:** Lars Michels, Philipp Stämpfli, Njoud Aldusary, Marco Piccirelli, Patrick Freund, Konrad P. Weber, Fabienne C. Fierz, Spyros Kollias, Ghislaine Traber

**Affiliations:** ^1^Department of Neuroradiology, Clinical Neuroscience Center, University Hospital Zurich and University of Zurich, Zurich, Switzerland; ^2^Department of Psychiatry, Psychotherapy and Psychosomatics, Psychiatric Hospital, University of Zurich, Zurich, Switzerland; ^3^Department of Diagnostic Radiology, Faculty of Applied Medical Sciences, King Abdulaziz University, Jeddah, Saudi Arabia; ^4^Balgrist University Hospital, Zurich, Switzerland; ^5^Department of Neurology, University Hospital Zurich and University of Zurich, Zurich, Switzerland; ^6^Department of Ophthalmology, University Hospital Zurich and University of Zurich, Zurich, Switzerland; ^7^Institute of Molecular and Clinical Ophthalmology Basel (IOB), Basel, Switzerland; ^8^Department of Ophthalmology, University of Basel, Basel, Switzerland

**Keywords:** diffusion-weighted imaging, visual snow, white matter, neuro-ophthalmology, inferior fronto-occipital fascicle

## Abstract

**Background:** Visual snow is considered a disorder of central visual processing resulting in a perturbed perception of constant binocular flickering or pixilation of the whole visual field. The underlying neurophysiological and structural alterations remain elusive.

**Methods:** In this study, we included patients (final *n* = 14, five dropouts; five females, mean age: 32 years) with visual snow syndrome (VSS) and age- and sex-matched controls (final *n* = 20, 6 dropouts, 13 females, mean age: 28.2 years). We applied diffusion tensor imaging to examine possible white matter (WM) alterations in patients with VSS.

**Results:** The patient group demonstrated higher (p-corrected < 0.05, adjusted for age and sex) fractional anisotropy (FA) and lower mean diffusivity (MD) and radial diffusivity (RD) compared to controls. These changes were seen in the prefrontal WM (including the inferior fronto-occipital fascicle), temporal and occipital WM, superior and middle longitudinal fascicle, and sagittal stratum. When additionally corrected for migraine or tinnitus—dominant comorbidities in VSS—similar group differences were seen for FA and RD, but less pronounced.

**Conclusions:** Our results indicate that patients with VSS present WM alterations in parts of the visual cortex and outside the visual cortex. As parts of the inferior fronto-occipital fascicle and sagittal stratum are associated with visual processing and visual conceptualisation, our results suggest that the WM alterations in these regions may indicate atypical visual processing in patients with VSS. Yet, the frequent presence of migraine and other comorbidities such as tinnitus in VSS makes it difficult to attribute WM disruptions solely to VSS.

## Introduction

Visual snow is a neurological state, defined by the presence of a continuous and chronic visual disturbance in the form of innumerable small dots covering the whole visual field (1). Patients with visual snow syndrome (VSS) experience a multi-layered array of visual symptoms in addition to the static itself, such as palinopsia, entoptic phenomena, nyctalopia and photophobia (2, 3). Visual snow denotes a spectrum type disorder that at its worse manifests with most of these additional symptoms, as well as with comorbidities such as migraine and tinnitus (4). In such instances, the condition is perceived as highly disabling (5). Though the pathophysiology of VSS remains largely indefinite (6) recent studies have provided some insight on the possible biological mechanisms underlying the condition. Behavioural (7) and neurophysiological studies (8, 9) have demonstrated patterns of changes indicating to increased cortical excitability and visual cortex dysfunction.

Through neuroimaging, it has been possible to reveal that VSS is characterised by altered metabolism of the extrastriate visual cortex (10–13). Recently, it has been shown by resting-state functional magnetic resonance imaging (fMRI) that VSS show hyperconnectivity (compared to healthy controls) between regions of the visual cortex but also in frontal, parietal and temporal brain regions (10, 12). In addition, task-based fMRI, electroencephalography as well as MR spectroscopy pointed towards an alteration in the visual and prefrontal (insular) cortex (8, 11).

Furthermore, it is known that VSS demonstrate structural, i.e., grey matter volumes, changes involving the visual system, and further expanding beyond it (13, 14). A consistent finding is that patients with VSS show increased grey matter volume of the lingual gyrus (10, 14).

Diffusion tensor imaging (DTI) is one method to assess white matter (WM) alterations on the microstructural level. In patients with migraine—a frequent comorbidity in VSS—alterations have been reported in fractional anisotropy (FA), mean diffusivity (MD), radial diffusivity (RD), and axial diffusivity (AD). For example, FA—an indirect surrogate of neuronal integrity—is lower compared to controls in migraineurs in multiple WM regions (15–18). So far, no study has yet examined if patients with VSS display WM abnormalities. Based on the previous (and above-described) studies on structural MRI, we hypothesise to see altered WM integrity in patients with VSS.

## Methods

### Sample

Inclusion criteria: 15 patients over 18 years of age and meeting the diagnostic criteria for VSS (1, 19) were recruited consecutively at the Department of Ophthalmology, University Hospital Zurich, Switzerland.

Exclusion criteria for all participants were pregnancy, presence of a neurodegenerative disorder, and contraindication against an MRI examination. The patients were all examined by senior neuro-ophthalmologists and neurologists. Patients were age and sex matched to 20 healthy controls (HCs). In both patients and HCs, the history was completed with regard to symptoms and conditions associated with VS syndrome as shown in Table 1.

**Table 1:** Summary of demographic and clinical values for patients with VSS.

			**Non-visual symptoms**							**Visual symptoms**					
**Patients**	**Age (years)**	**Sex**	**Migraine**	**With aura**	**Tinnitus**	**Depression**	**Anxiety**	**Duration of VS (years)**	**Imbalance**	**Palinopsia**	**Blue field entoptic phenomenon**	**Other entoptic phenomena**	**Photophobia**	**Glare**	**Nyctalopia**
P1	44	0	1	0	1	0	0	9.0	1	0	0	1	0	0	1
P2	47	1	1	1	1	1	0	17.0	1	0	1	0	0	1	1
P3	23	1	0	0	0	1	0	5.5	0	0	1	0	0	0	1
P4	33	1	0	0	1	0	1	4.2	0	0	0	1	1	1	0
P5	18	1	0	0	1	1	1	1.0	1	1	1	0	1	0	1
P6	19	1	0	0	1	0	0	19.3	0	0	0	1	0	0	0
P7	44	0	1	1	0	1	1	4.1	0	1	0	0	1	1	1
P8	30	0	1	1	1	0	0	4.9	0	1	0	0	1	0	0
P9	39	0	1	1	1	0	1	0.8	0	0	1	0	1	1	1
P10	33	1	0	0	0	0	0	2.0	1	0	1	1	1	0	0
P11	21	1	1	1	1	0	0	1.2	1	1	0	1	0	1	0
P12	54	0	1	1	1	1	0	0.6	0	1	1	1	1	0	0
P13	22	1	0	0	1	0	0	6.0	0	0	0	0	0	1	1
P14	30	1	0	0	0	0	0	5.0	0	0	1	1	0	0	1

*For sex, 1 = male, 0 = female. For all other variables, 1 = present, 0 = absent. Six of seven patients with migraine demonstrated visual aura*.

The following clinical measures were included: duration of VS symptoms, history of migraine, tinnitus, anxiety, depression, tremor or imbalance, and perception of palinopsia, blue field entopic phenomena, other entoptic phenomena, photophobia, glare, nyctalopia, symptoms in darkness, symptom presence with eyes closed, and overall perceived symptom severity on a scale of 0–10. Migraine occurrence was assessed with the Diagnostic Algorithm of the Hardship Questionnaire (20). Participants were asked whether they had been diagnosed with, or feeling they were suffering from, an anxiety disorder or depression but no patients indicated the presence of anxiety or depression. None of the VS patients showed any signs of an underlying ophthalmic pathology based on the history and the clinical examination including best corrected visual acuity, static perimetry (Octopus 900, Haag-Streit, Bern, Switzerland), fundoscopy, and optical coherence tomography of the macula and the peripapillary retinal nerve fibre layer (Heidelberg Spectralis, Heidelberg Engineering, Heidelberg, Germany). All subjects provided informed written consent to participate in this study, which was approved by the ethics committee (Canton Zurich, Switzerland, BASEC-NR: 2016-00225).

### MRI Data Acquisition

MRI data acquisition was performed on a 3T whole-body MR scanner (Ingenia, Philips Healthcare, Best, the Netherlands), equipped with 80 mT/m gradients and a 32-channel receive head coil. Diffusion data were acquired using a diffusion-weighted single-shot spin-echo echo-planar-imaging sequence with the following parameters: repetition time (TR): 9,837 ms, echo time (TE): 94 ms, field of view (FOV): 224 × 224 mm^2^, 55 contiguous transversal slices, slice thickness: 1.7 mm, acquisition matrix: 132 × 130, SENSE factor: 2, partial Fourier encoding 68%. The bounding box was planned with having the inferior slice positioned at the inferior border of the cerebellum, defined on a T1-weighted midline sagittal survey image. Due to the small slice thickness, we did not cover the whole-brain but only included regions inferior to the body of the corpus callosum (covering the corpus callosum as well).

Diffusion acquisition was performed along 128 directions with a b-value of 1,000 s/mm^2^ and two signal averages. Additionally, one non-diffusion-weighted b = 0 s/mm^2^ scans were acquired resulting in a scan time of 21 min 40 s. For structural reference and anatomical priors for the tracking algorithm, T1-weighted images were recorded using a three-dimensional magnetisation prepared rapid gradient-echo (MP-RAGE) sequence with 1 mm isotropic resolution.

### Diffusion Data Pre-processing

Before any pre-processing steps, quality control of all acquired diffusion data was assessed based on several criteria: First, diffusion tensor residuals were calculated for every acquired diffusion direction and the nine slices in the whole diffusion dataset with the highest residuals were identified for visual inspection. Plots were generated depicting the 12 slices (four sagittal, four axial, and four coronal directions) with the highest noise level. Second, mean signal intensity plots for every diffusion direction and the non-diffusion-weighted image were derived and plotted slice by slice in sagittal, axial, and coronal directions. Artefacts, such as signal dropouts due to head motion, can easily be spotted on these plots. A trained MR physicist inspected the data for artefacts and rated the signal courses and fitting residuals of every subject on a Likert-type scale.

Pre-processing diffusion data followed a similar procedure previously described in our recent publication (21). After denoising the raw data using the “dwidenoise function” from the MRtrix3 software package (https://www.mrtrix.org/), diffusion weighted data were first corrected for eddy-current and motion induced distortions by registration the diffusion weighted images to the b0 image using the dwipreproc routine from MRtrix3 software package. This function makes use of the eddy tool implemented in FSL (FMRIB, Oxford, UK version 6.0.0) (22). The brain extraction tool (BET) from FSL was then applied to remove non-brain tissue and estimate the inner- and outer skull surfaces. Next, the diffusion data were corrected for susceptibility-induced distortions using the “bdp correction algorithm” implemented in the BrainSuite software package (http://brainsuite.org) (23). Diffusion maps derived from the diffusion tensor, i.e., FA, MD, RD, and AD were then calculated using the DTIFIT tool implemented in the FSL software package (https://fsl.fmrib.ox.ac.uk/fsl/fslwiki).

### Statistical Analysis

To evaluate differences between the groups, voxel-wise (whole-brain) Tract-Based Spatial Statistics (TBSS, https://fsl.fmrib.ox.ac.uk/fsl/fslwiki/TBSS) analysis based on a general linear model was performed using FSL's randomise tool (24) with 5,000 permutations to correct for multiple comparisons (*p* < 0.05, corrected). All results included threshold-free cluster enhancement (TFCE) (25). Three statistical contrasts were computed, testing for positive and negative differences of the DTI parameters between the patients with VSS and HCs:

- General linear model, with correction for age and sex (i.e., age and sex were used as nuisance variables in the model).
- General linear model, with correction for age, sex, and migraine occurrence
- General linear model, with correction for age, sex, and tinnitus occurrence.

## Results

### Demography and Clinical Data

Seven patients showed episodic migraine; six of them demonstrated visual aura (Table 1 provides a summary of demographic and clinical data for the VSS group). Based on the HARDSHIP questionnaire, three HCs showed migraine, and were thus excluded. Groups did not differ in sex (*p* = 0.14, Chi-Square test) or age (*p* = 0.13, unpaired *t*-test; mean age VSS group: 32.6 ± 11.1 years, mean age HCs: 28.2 ± 5.5 years).

### Image Quality

Five patients and three HCs had to be excluded because of poor DTI data quality (strong head motion resulting in artefacts on the FA map). Hence, the reported results are based on 14 patients with VSS and 20 HCs.

### DTI Findings

**Figure 1:**
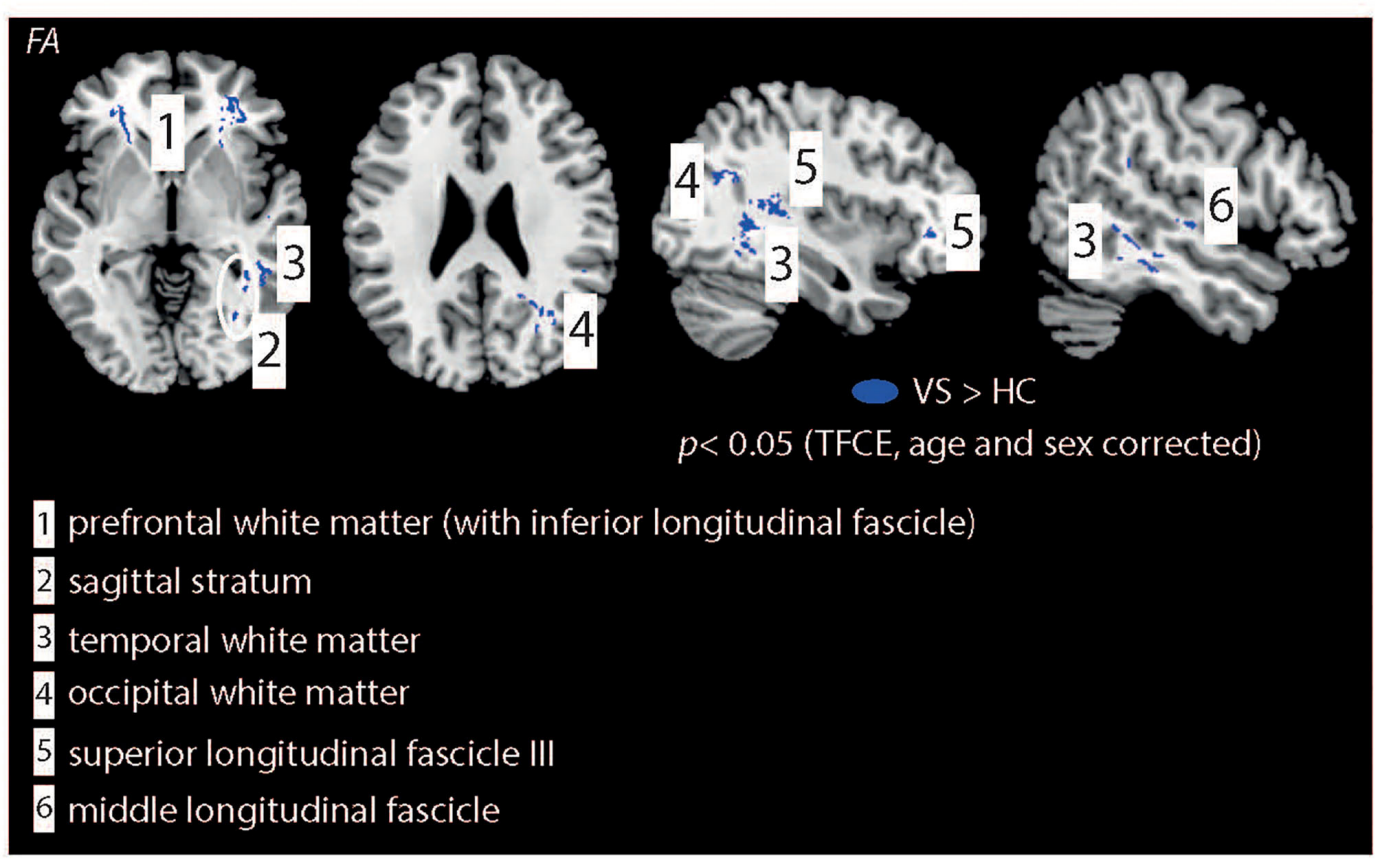
Illustration of WM changes for the statistical comparison “HCs vs. patients with VSS.” Patients demonstrated higher FA values in multiple brain regions. In contrast, HC showed higher MD and RD values compared to patients with VSS (not shown, see Table 2). All results are TFCE, age and sex corrected. IFOF, Inferior Fronto-Occipital fascicle.

The VSS patient group demonstrated higher (p-corrected < 0.05, adjusted for age and sex, Figure 1) FA, lower MD and RD values compared to HCs. FA changes were seen in the prefrontal WM [with extension into the inferior fronto-occipital fascicle (IFOF)], sagittal stratum, temporal and occipital WM, superior longitudinal fascicle (SLF3), and middle longitudinal fascicle. For MD changes were additionally seen in the corpus callosum (genu) but not in the sagittal stratum, middle longitudinal fascicle and occipital WM. No significant group differences were seen for AD.

**Figure 2:**
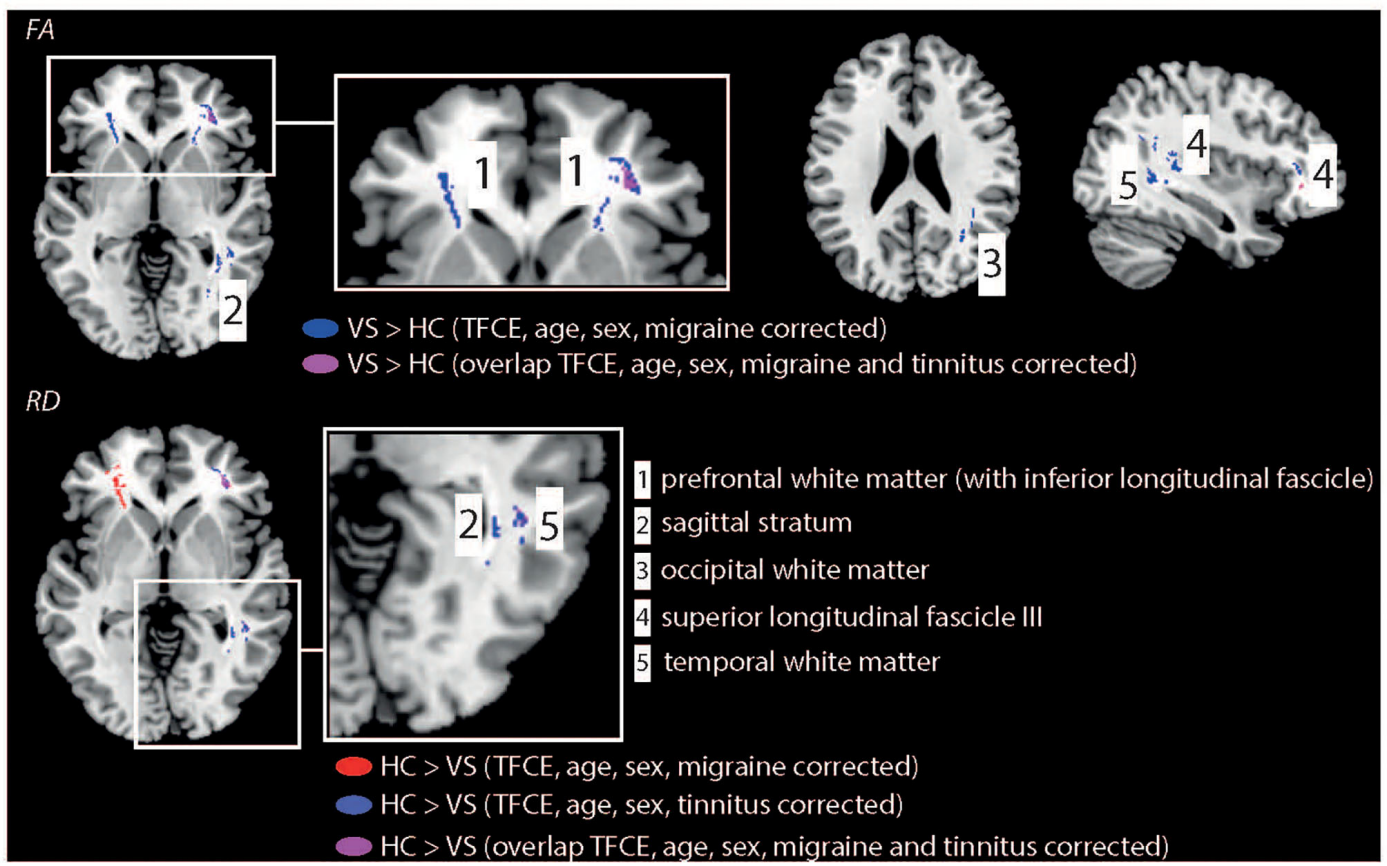
Illustration of WM changes for the statistical comparison “HCs vs. patients with VSS.” Patients demonstrated higher FA values in multiple brain regions. In contrast, HC showed higher RD values compared to patients with VSS. Results were TFCE corrected and corrected for age, sex, and additionally for migraine or tinnitus.

When additionally corrected for interictal migraine occurrence (Figure 2), FA changes were seen in the same WM areas as well except of the middle longitudinal fascicle. RD changes were seen in the prefrontal WM (with extension into the IFOF), right SLF3, and temporal WM. Yet, no significant group differences were observed for MD and AD. When additionally corrected for tinnitus occurrence FA were only seen in the right prefrontal WM, SLF3, and sagittal stratum. For RD changes were observed in the right prefrontal WM (with extension into the IFOF), SLF3, temporal and occipital WM, and sagittal stratum. Table 2 shows the full summary of WM group differences.

**Table 2:** Summary of DTI group differences.

**Measure**	**Direction**	**Region**	**Hemisphere**
**A)**			
**DTI**			
FA	*VSS > HC*		
		Prefrontal WM (with inferior fronto-occipital fascicle)	Bilateral
		Superior longitudinal fascicle III	Right
		Occipital WM	Right
		Temporal WM	Right
		Middle longitudinal WM	Right
		Sagittal stratum	Right
MD	*HC > VSS*		
		Prefrontal WM (with inferior fronto-occipital fascicle)	Bilateral
		Superior longitudinal fascicle III	Right
		Corpus callosum	n.a.
		Temporal WM	Right
RD	*HC > VSS*		
		Prefrontal WM (with inferior fronto-occipital fascicle)	Bilateral
		Superior longitudinal fascicle III	Right
		Occipital WM	Right
		Temporal WM	Right
		Middle longitudinal WM	Right
		Sagittal stratum	Right
**B)**			
**DTI**			
FA	*VSS > HC*		
		Prefrontal WM (with inferior fronto-occipital fascicle)	Bilateral
		Superior longitudinal fascicle III	Right
		Occipital WM	Right
		Temporal WM	Right
		Sagittal stratum	Right
RD	*HC > VSS*		
		Prefrontal WM (with inferior fronto-occipital fascicle)	Bilateral
		Superior longitudinal fascicle III	Right
		Temporal WM	Right
**C)**			
**DTI**			
FA	*VSS > HC*		
		Prefrontal WM (with inferior fronto-occipital fascicle)	Right
		Superior longitudinal fascicle III	Right
		Sagittal stratum	Right
RD	*HC > VSS*		
		Prefrontal WM (with inferior fronto-occipital fascicle)	Right
		Superior longitudinal fascicle III	Right
		Occipital WM	Right
		Temporal WM	Right
		Sagittal stratum	Right

*HC, healthy controls; VSS, patients with visual snow syndrome; n.a., not applicable. A) Results are TFCE, age, and sex corrected. B) Results are TFCE, age, and sex as well as for migraine. C) Results are TFCE, age, and sex corrected as well as for tinnitus*.

## Discussion

Our study demonstrated widespread WM alterations in patients with VSS. We thus add to the growing body of literature reporting structural, i.e., grey matter volume, abnormalities in the visual cortex and visual association cortex. A novel finding is that structural WM alterations are evident in the visual cortex but also in the frontal and temporal cortex. Group differences were similar but less strong compared to the analysis without correcting for migraine or tinnitus. Consistent changes for both analyses were seen in the IFOF, sagittal stratum and right SLF. We suggest that these abnormalities could thus be associated to the manifestation of VS rather than by the presence of migraine.

Parts of the IFOF are associated with visual processing (26) by visual conceptualisation (27) and visual hallucinations (28, 29). Our results suggest that the WM alterations in these regions might indicate atypical visual processing in patients with VSS. Similarly, Aldhafeeri et al. found a disruption of WM integrity in the IFOF in patients suffering from tinnitus, a frequent comorbidity in individuals affected by VSS (30). Yet, even after correction of tinnitus presence, alterations were seen in the right prefrontal WM (IFOF). This suggests that this region might therefore be directly involved in the underlying biology of the condition.

The SLF is involved in speech processing (31, 32), musical processing (33), spatial attention (34) and memory (35), decision making (36), visual perception (37), and perceptual organisation (38). For example, right-hemisphere brain damage e.g., induced by stroke often results in visual-spatial deficits, such as a neglect (37). McKendrick et al. (7) demonstrated that patients with visual snow demonstrated reduced centre-surround contrast suppression and elevated luminance increment thresholds in noise but did not differ on a global form or global motion task. Our study suggests that patients with visual snow may show not only deficits in visual perceptual measures involving the suprathreshold processing of contrast and luminance but also in tasks involving high-order visual brain regions. Yet, this needs to be verified by psychophysical testing combined with structural neuroimaging (such as DTI).

Alterations in patients with VSS were also seen in the sagittal stratum, which contains the IFOF, inferior longitudinal fasciculus, and posterior thalamic radiation (39–41). Specifically, the sagittal stratum is a major cortico-subcortical WM bundle that conveys fibres from the parietal, occipital, cingulate, and temporal regions to subcortical destinations in the thalamus, pontine nuclei, and other brainstem structures (42). It additionally has afferents from the thalamus to the cortex, thus, it is a major subcortical fibre system and not exclusively a fibre tract linking the lateral geniculate nucleus with the calcarine cortex. Recently, electrical stimulation in patients undergoing wide-awake surgery for a cerebral glioma was applied combined with behavioural tasks (including visual and somesthetic processes, semantics as well as language, spatial and social cognition) to monitor online the patients' functions during stimulation (43). Stimulation of the right sagittal stratum lead to visual disturbances, visual hemi-agnosia, semantic paraphasia, left spatial neglect, confusion and comprehension difficulties, anomia, and mentalizing disturbances. We suggest that the observed DTI alterations in this region could be associated with some of the known visual disturbances generally observed in patients with VSS.

The alterations in temporal WM regions parallel findings of our recent resting-state fMRI connectivity study, performed in a similar sample of patients and controls (10). The middle and superior temporal cortex are involved in object, motion and form processing (44, 45) and abnormal WM could point towards a disturbed information processing in patients with VSS in these regions.

In general, we found stronger FA values for patients. Therefore, our data could indicate that patients demonstrate elevated excitability of parts of the visual cortex as well as other brain regions. Yet, further research is required to provide a more direct evidence for this proposed mechanism. We observed that WM impairments showed a right-hemispheric lateralisation (e.g., right IFOF), when results were corrected for the presence of tinnitus. This extends previous functional PET studies, who reported metabolic alterations in the right visual (lingual) gyrus (13, 19). However, the origin of the tentative anatomical lateralisation has not been examined in detail and further studies are needed to replicate this observation, especially examining larger cohorts of patients with VSS with and without tinnitus. In contrast to structural (VBM or DTI) studies, resting state fMRI studies reported abnormal functional connectivity in visual snow patients in both hemispheres (10, 12).

The ability to measure perceptual parameters in visual snow reveals promise for the development of novel ancillary tests. They may help to assist in visual snow diagnosis and possibly as a method for quantitatively assaying any benefits of treatment.

## Limitations

The lack of whole-brain coverage is a strong limitation of our study. Hence, we could not examine if WM alterations might be present e.g., in regions superior of the corpus callosum, e.g., in the parietal cortex, superior frontal regions, somatosensory, or (pre-)motor cortex. Future studies should be performed to examine this question in full detail. New DTI measures with clinical relevance, such as fibre density (46), could be additionally explored in upcoming studies. Regarding migraine presence in HCs, we excluded (based on the Hardship questionnaire) all subjects with migraine. In addition, there is no validated genetic marker (in contrast to e.g., Alzheimer's disease) for migraine. For patients, we did no assess—e.g., by headache diaries—the presence of a migraine attack the day before or the days after scanning. Thus, it might be that patients (with migraine) were scanned in an acute pre- or postictal phase.

## Data Availability Statement

The raw data supporting the conclusions of this article will be made available by the authors, without undue reservation.

## Ethics Statement

The studies involving human participants were reviewed and approved by Ethics Committee Canton Zurich (Switzerland), BASEC-NR: 2016-00225. The patients/participants provided their written informed consent to participate in this study.

## Author Contributions

LM did the statistical analysis and wrote the first manuscript draft. PS helped with data post-processing. LM, GT, MP, and SK were involved in the study design. NA did the data recording and helped with data analysis. FF, GT, and KW helped with patient recruitment, clinical interviews, and paper writing. MP set up the MRI sequences and helped with DTI scanning. All authors helped with paper writing.

## Funding

NA received financial support from the Saudi Arabian Cultural Mission (Paris, France). PF was funded by an SNF Eccellenza Professorial Fellowship grant (PCEFP3_181362/1). GT (2016–2018) and FF (2020–2021) received a grant from the career development program Filling the Gap, Faculty of Medicine, University of Zurich.

## Conflict of Interest

The authors declare that the research was conducted in the absence of any commercial or financial relationships that could be construed as a potential conflict of interest.

## Publisher's Note

All claims expressed in this article are solely those of the authors and do not necessarily represent those of their affiliated organizations, or those of the publisher, the editors and the reviewers. Any product that may be evaluated in this article, or claim that may be made by its manufacturer, is not guaranteed or endorsed by the publisher.
